# Implications of Isomorphism in the Family of Apatite Compounds

**DOI:** 10.3390/ijms26094397

**Published:** 2025-05-06

**Authors:** Agnieszka Lasota, Mieczysław Gorzelak, Emanuela Bis, Przemysław Biliński, Krzysztof Gieburowski, Wojciech Kłapeć, Barbara Tymczyna-Borowicz, Michał Łobacz, Jarosław Pawlicz, Maciej Jarzębski, Marek Wieruszewski, Karolina Turżańska, Mirosław Jabłoński, Andrzej Kuczumow

**Affiliations:** 1 Department of Jaw Orthopaedics, Medical University of Lublin, 20-093 Lublin, Poland; agnieszka.lasota@umlub.pl; 2 Department of Orthopaedics and Rehabilitation, Medical University of Lublin, 20-059 Lublin, Poland; b.leszczynska@umlub.pl (M.G.); wklapec@gmail.com (W.K.); karolina.turzanska@umlub.pl (K.T.); miroslaw.jablonski@umlub.pl (M.J.); 3 Student Research Group at the Chair and Department of Oral Medicine, Medical University of Lublin, Chodźki 6, 20-093 Lublin, Poland; emanuelabis333@gmail.com; 4 Faculty of Medicine and Health Promotion, The President Stanisław Wojciechowski University of Kalisz, 62-800 Kalisz, Poland; bildom@gmail.com (P.B.); k.gieburowski@uniwersytetkaliski.edu.pl (K.G.); 5 Department of Conservative Dentistry with Endodontics, Medical University of Lublin, 20-093 Lublin, Poland; barbara.tymczyna-borowicz@umlub.pl; 6 Department of Oral Surgery, Medical University of Lublin, Chodźki 6, 20-093 Lublin, Poland; michal.lobacz@umlub.pl; 7 Department of Orthopedics and Traumatology, Poznan University of Medical Sciences, 28 Czerwca 1956 135/147, 61-545 Poznań, Poland; jarek.pawlicz@gmail.com; 8 Department of Physics and Biophysics, Poznań University of Life Sciences, 60-637 Poznań, Poland; 9 Department of Wood-Based Materials, Faculty of Forestry and Wood Technology, Poznań University of Life Sciences, Wojska Polskiego 28, 60-627 Poznań, Poland; marek.wieruszewski@up.poznan.pl

**Keywords:** isomorphism, apatite, energy of ion exchange substitutions, cations, anions, channel ions

## Abstract

Apatites are very important compounds of mineralogical and biological meaning. Apatites originated from the calcium hydroxy compound 3Ca_3_(PO_4_)_2_·Ca(OH)_2_ and potentially might form three series of isomorphic salts, derived from cationic substitutions in the positions of Ca(I) and Ca(II) ions in the core compound; anionic substitutions of phosphates; and substitutions of anions and very simple chemical entities instead of the hydroxyl group in channel locations. The energies coupled with the ion exchanges inside those three locations were studied using our original method resulting from the transformation of Braggs’ law. The energy changes resulting from the ion exchanges were studied in connection with either the ionic radii for the cations or ionic volumes for the anions. The same series were observed when the variabilities of energy were confronted with the variabilities in the sinus of diffraction angle Θ showing changes in momentum transfer. In particular, the relationships between the energy changes and the coupled changes in the universal crystallographic parameter d showed the surprising uniformity of all ion exchanges in the apatites. The incremental change in the Braggs’ d-parameter always demands the same change in the energy, with good approximation, independently of the location of the ion exchange. So, the isomorphism of the apatites is not triple but a uniform one at the energy level. Such an approach enables the estimation of the volume of the ion-□ (□-vacancies) agglomerates. The introduction of ions with greater volumes exerts the phenomenon of swelling of apatite cells, which can be quantitatively estimated. The dependence of diffraction spectra on the temperature allows for the determination of minimal values of crystallographic cell volumes and d parameters at the temperature of 0 K. In sum, the study of energies connected with the change of Bragg dimension d is a new and valuable method of insight into the behaviour of apatite crystals.

## 1. Introduction

Isomorphic substances are compounds that possess uniform crystallographic structures with nonidentical but coupled dimensions. In theory, their structures might be imposed from the first substance to the next one with a small correction of the parameters. For that reason, they can form solid solutions with other members of the same isomorphic series, e.g., if it concerns the apatite the apatite series [1]. Apatites often exist in nature, and some classes of them serve as the biomaterials for hard tissues of all the vertebrates. There are the modified carbonated as well as magnesium- and sodium-involving hydroxyapatites with the specific name bioapatites. In this sense, apatites are important both in the mineralogical/geological meaning of forming some important rocks [2] and also in biological and medical practice [3,4]. They are worth attention since one can trace the similarities and differences between them and also observe the mutual transitions. The compounds belonging to the same family of isomorphic species form the series [5]. Some empirical rules, such as that one by Goldschmidt [6,7], indicate what kind of ions can substitute the original ions in the molecule. The ion exchange process depends on the electrostatic interaction between entering ions and the spatially arranged components of the crystallographic network. The spatial obstacles are the other factors influencing the reaction. The microporous structure of the apatite particles, containing the channels, makes the ion exchanges easier [8]. The mentioned substitutions can be treated as the reactions leading to the synthesis of the new compounds, but in everyday practice, they can serve to capture the metallic components of the soils and wastes [9], even radioactive ones [10]. Very important ion exchanges with the selected elements occur in bioapatites in hard tissues [11,12], leading both to erosion [13,14] and repairing the tissues [15,16] in different cases [17]. The processes of maturation and aging of the hard tissues [18] are also related, at least partially, to the ion exchanges. In this contribution, we will consider only the mineral-type apatite series of isomorphic compounds. We studied earlier the apatites and bioapatites as members of the simple isomorphic series [19]. Still, it seems that something can be added to the knowledge of the variability inside the apatite series. This variability results from the wide possibility of doping the structure [2]. The considerations will start with some remarks on the following mineralogical formula: 3Ca_3_(PO_4_)_2_ꞏCa(OH)_2_ (alternatively Ca_5_(PO_4_)_3_OH, and crystallography-oriented version Ca(2)_6_Ca(1)_4_(PO_4_)_6_(OH)_2_). Here, the anions from the channel position can be exchanged and just the most popular division of apatites into hydroxy-, fluor-, chlor-, and bromapatite is derived from these substitutions. Thus, one can form the relevant series of isomorphic substances. But we postulate that the next isomorphic series of apatites can be derived from the substitutions of phosphate ions with vanadate, arsenate, and chromate ions. The final and widest possibility can be executed by the substitutions of Ca^2+^ by metallic ions of the following elements: Mg, Sr, Ba, Cd, and Pb, to limit themselves only to elements that can be completely introduced. In such a situation we put the hypothesis that apatite should form three different series of isomorphic substances, depending on the attacked part of the particle. In our earlier studies, we managed to link the chemical changes with the energies necessary to perform them. It is a quite powerful tool to trace the chemical variability of apatites. Using the methodology elaborated in [19], we should check if those series differ mutually and—if so—to which degree.

In this paper, we focused on the following aims: i. checking the above hypothesis on mineral apatites, ii. consideration of swelling of apatites due to ion exchanges with greater ions, iii. paying attention to the behaviour of vacancies, iv. consideration of consequences of heating apatites.

## 2. Results

### 2.1. Checking the Triple Way of Isomorphism in Apatites

As the first, the cationic exchanges were investigated for the cations enumerated earlier. Of course, many other elements (e.g., Fe, Ni, Co) have a possibility to enter the apatite structure, but they do it incompletely. The data, cited in Table 1, are divided into three groups, concerning the ion exchanges of either the main cations or main anions or ions in the channel locations. The last column indicates the bibliography position, from which we have taken the data. As one can see, the data are from very different, independent, and credible sources.

**Figure 1:**
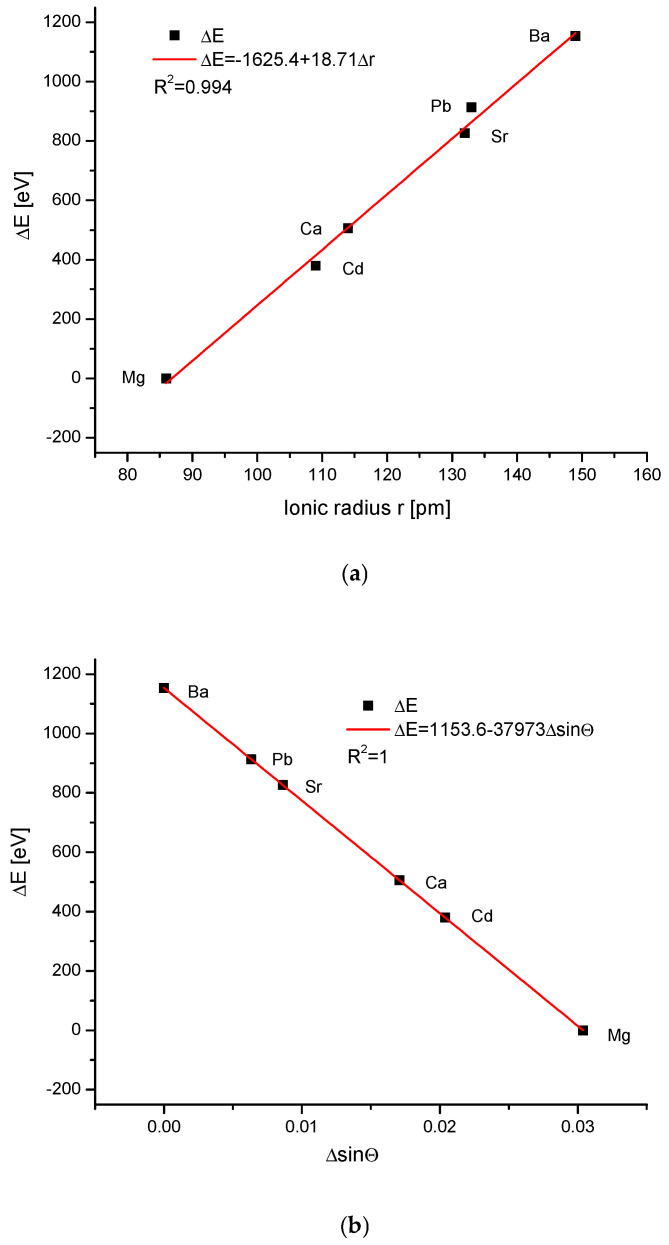

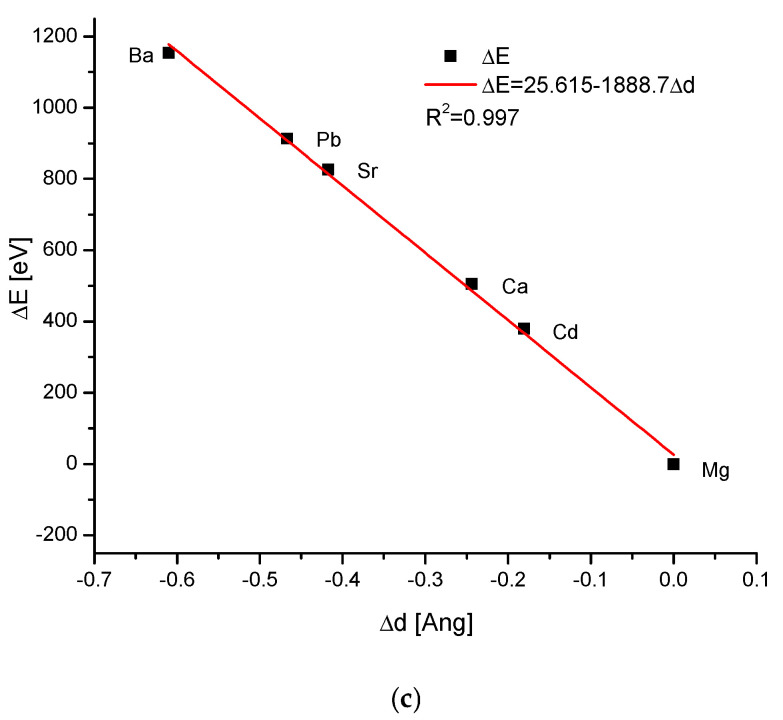
(**a**) The relationship between the change of the energy during the cationic ion exchanges in the apatites and the value of ionic radii, with the latter taken from Shannon [34]; (**b**) the coupling between the energy changes and the difference in sinΘ values; (**c**) the dependence of the energy changes during the ion exchanges and the change in the universal crystallographic parameter d.

The energy increments calculated according to Equation (1) were linearly correlated with the magnitude of the ionic radii (Figure 1a). The result is even more impressive due to the fact that the results were totally independent of each other—the structural studies of apatites were made in different laboratories and by the use of totally different methods than the measurements and calculation of ionic radii. The latter were estimated as a rule from the thermochemical experiments and calculations. As we know from our earlier studies, such a linear relationship is by no means common in the case of different isomorphic series [19]. The linearity (or another simple functional relationship) together with the close to 1 value of the coefficient of determination R^2^ testify that the compound belongs to one family of apatites and to the same crystallographic system. The unavoidable scatter of results if they are supported by the data from different authors/laboratories (see column References in Table 1) transformed in very small shifts on the curves. The shifts were so small that they did not disturb the natural order of the elements resulting from the Periodic Table. The dependences of the energy changes against the changes in the values of sinΘ (Figure 1b) and d (Figure 1c), the universal crystallographic parameter, respectively, were simple and elegant linear functions. The values of the coefficients of determination, extremely close or even equal to 1, testify that we can indeed talk about the functional relationships. The passing from the lightest element Mg to the last one Ba resulted in the decrease in values of Δd, and each increment of Δd corresponded to the relevant positive increment of ΔE.

**Figure 2:**
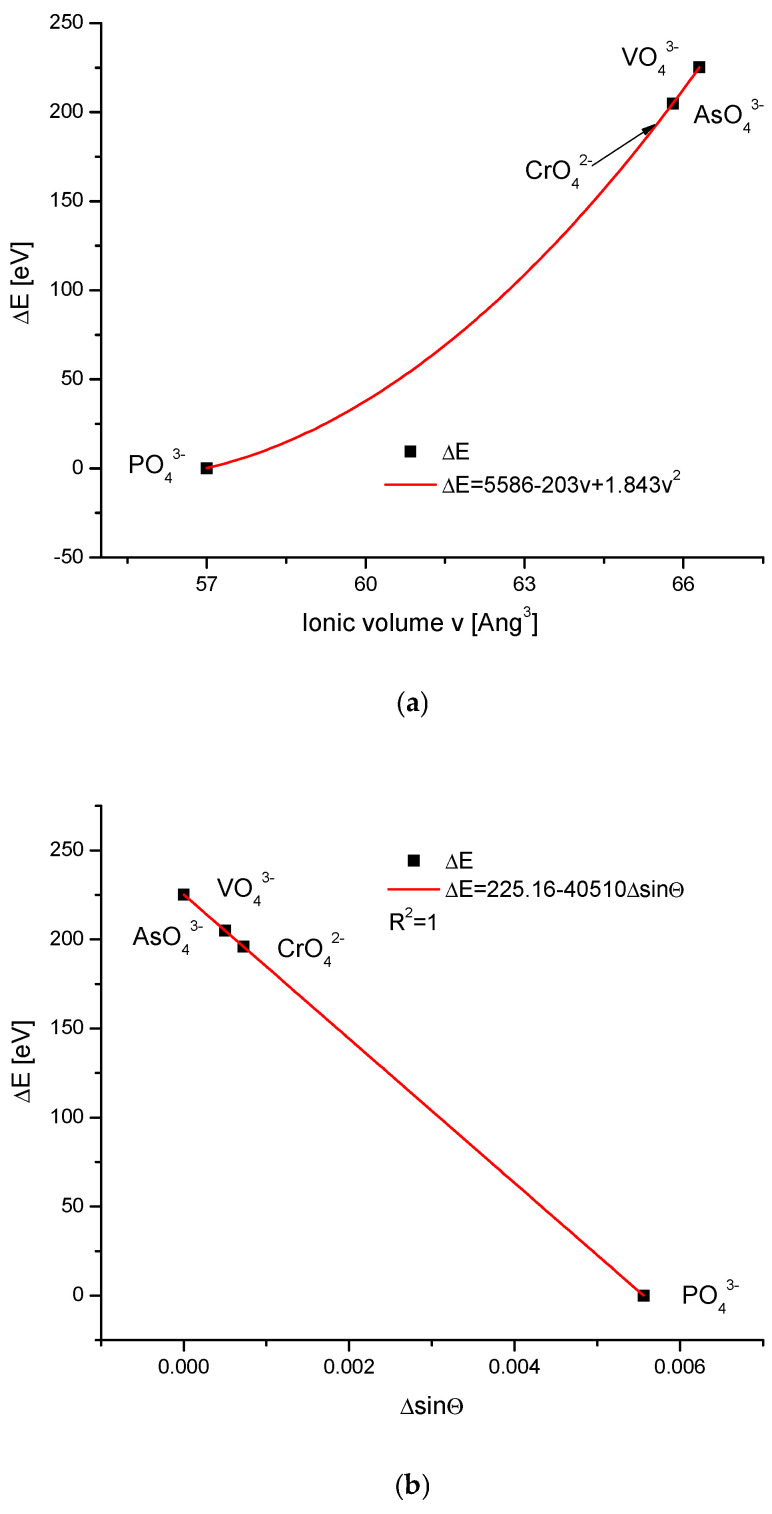

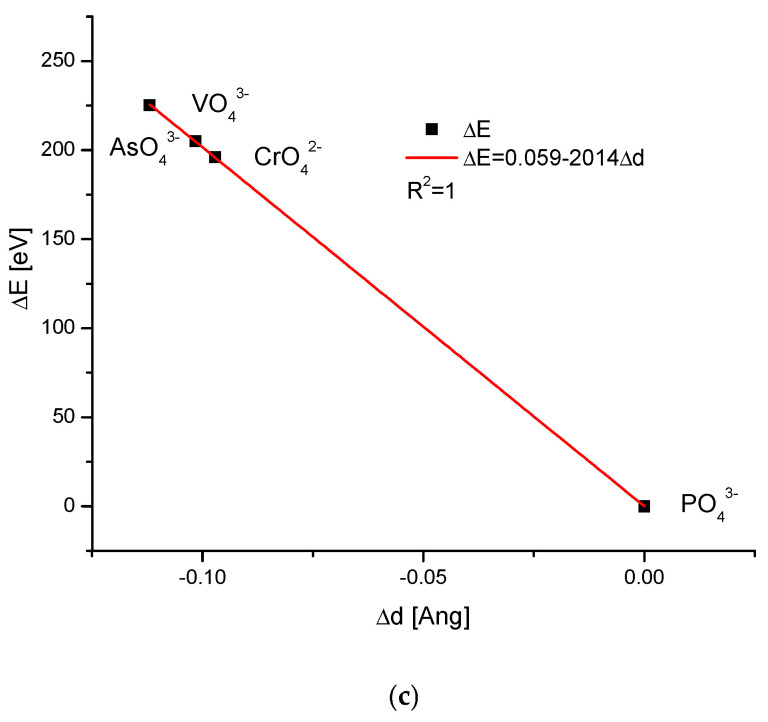
(**a**) The relationship between the change of the energy during the anionic ion exchange in the apatites and the anionic volumes, with the latter ones taken from Glasser and Jenkins [38]; (**b**) the coupling between the energy changes and the difference in the sinΘ values); (**c**) the dependence of the energy changes during the ion exchanges and the change in the universal crystallographic parameter d. The arrow in Figure 2a shows the postulated value of CrO_4_^2−^ ionic volume.

While the values of the ionic radii used in Figure 1 and the volumes of simple anions used in the further text, concerning the ion exchanges in the channel positions, did not generate serious doubts concerning the quality of the data and could be easily cross-checked, the values of complex oxoanions of the type XO_4_^n−^ were widely scattered, even in the publications by the same authors [35,36,37,38]. We decided to select the data from the position by Glasser and Jenkins [38] since it was the only option, which was consistent with the crystallographic relationships of the changes in the energy against the changes in the values of sinΘ and the universal crystallographic parameter d; otherwise, these data seemed to be most reasonable. The two latter diagrams in Figure 2 present the regular, functional (linear) relationships, similar as for the cations. The dependencies of energy changes on the ionic volume obeyed the polynomial quadratic function. All that implies the value of the volume of CrO_4_^2−^ as ~65Å^3^. This value was missing in the data by Glasser and Jenkins [38], and was very variable in other publications. The polynomial relationship indicates that the anionic ion exchanges were more complicated than their cationic counterparts. A great difference was observed between the data concerning the phosphates and all other heavier anions.

**Figure 3:**
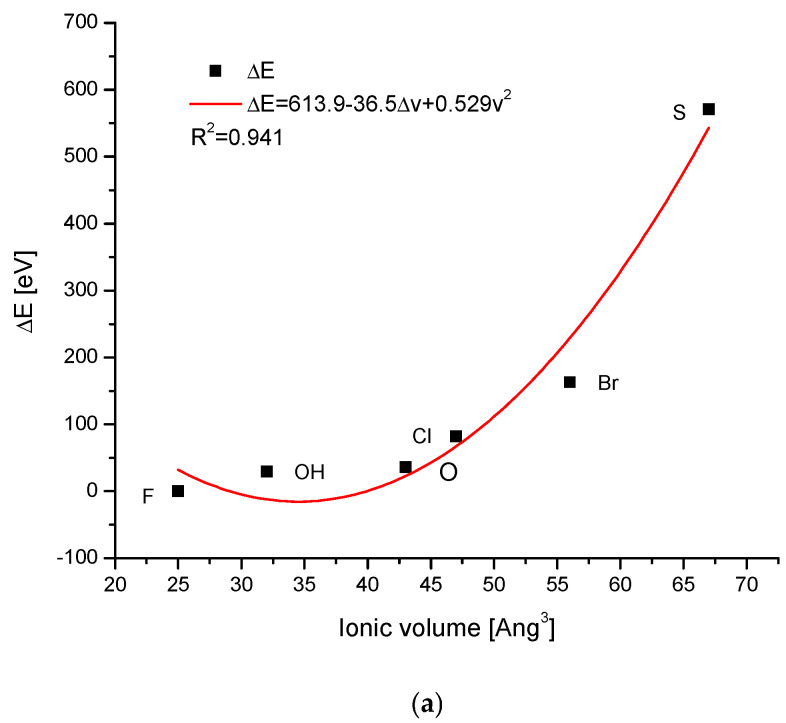

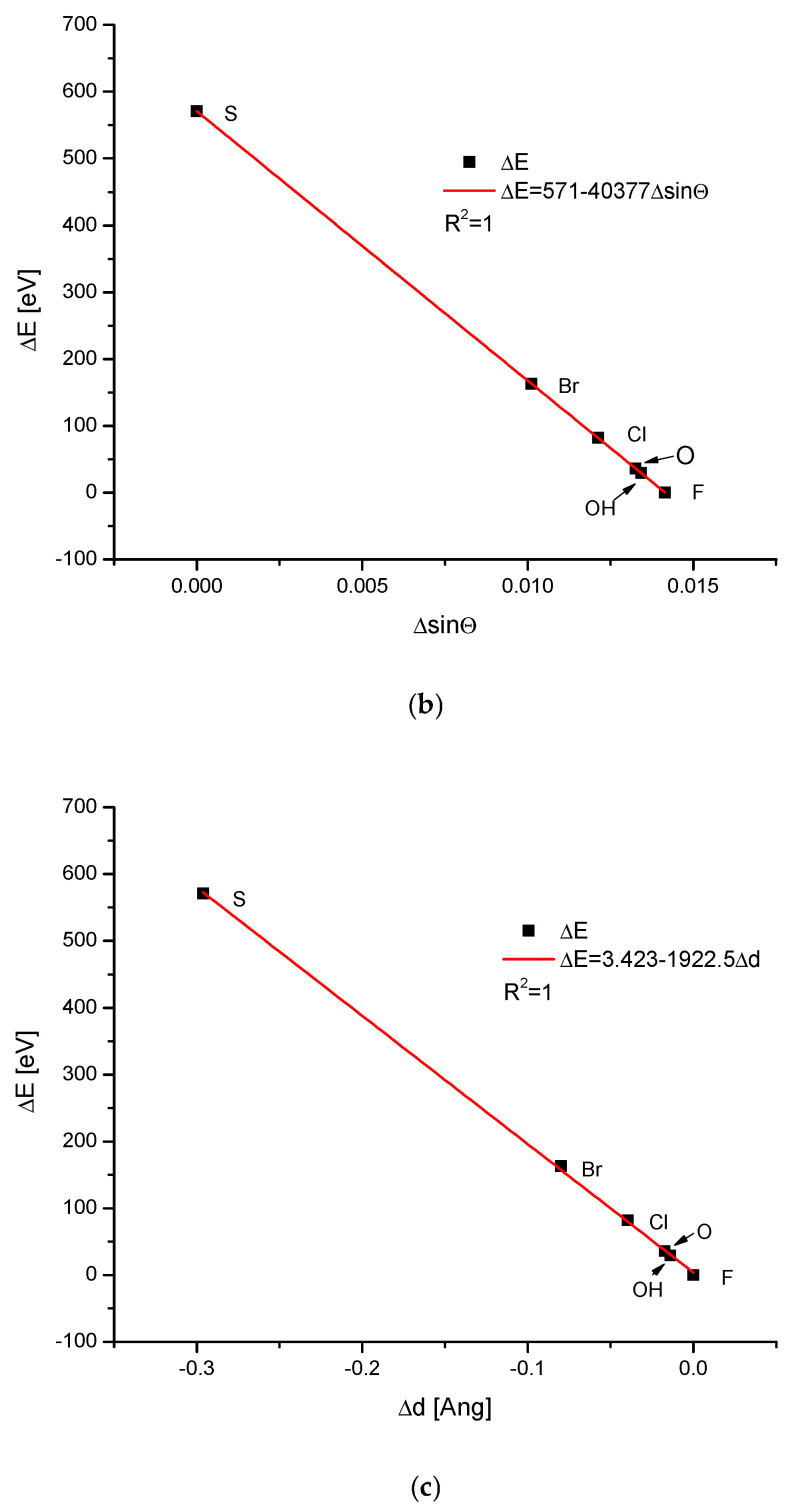
(**a**) The relationship between the change of the energy during the anionic ion exchange in the channel locations and the ionic volume of channel anions, with the latter taken from Jenkins et al. [35] (**b**) the coupling between the energy changes and the difference in the sinΘ values; (**c**) the dependence of the energy changes during the ion exchanges and the change in the universal crystallographic parameter d.

The ion exchanges in the channel positions involved many interesting anions. The dependence of the energy changes on the ionic volumes was quite regular, being the polynomial of the second degree, with somewhat greater scattering than in the previous cases (Figure 3a). The relationships of the changes in the energies during the ion exchanges against the changes in the sinΘ or d parameter were linear (Figure 3a,b), as in all previous cases. It is worth noting that on the first curve, the observed distance of ionic radii between OH^−^ and O^2−^ ions was relatively large, while the change in the energy of ion exchange was very small. On the two remaining diagrams, the differences between the two mentioned ions were hardly noticeable. The sulphur version of the apatite diverged very seriously from other species but still belonged to the same family of isomorphic substances.

### 2.2. Swelling of Apatites Due to Ion Exchanges

**Figure 4:**
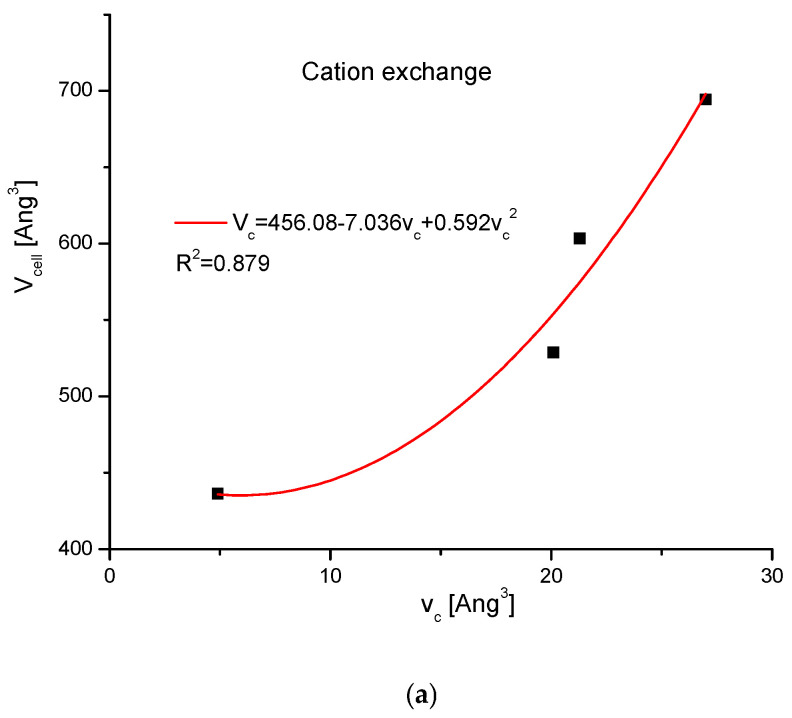

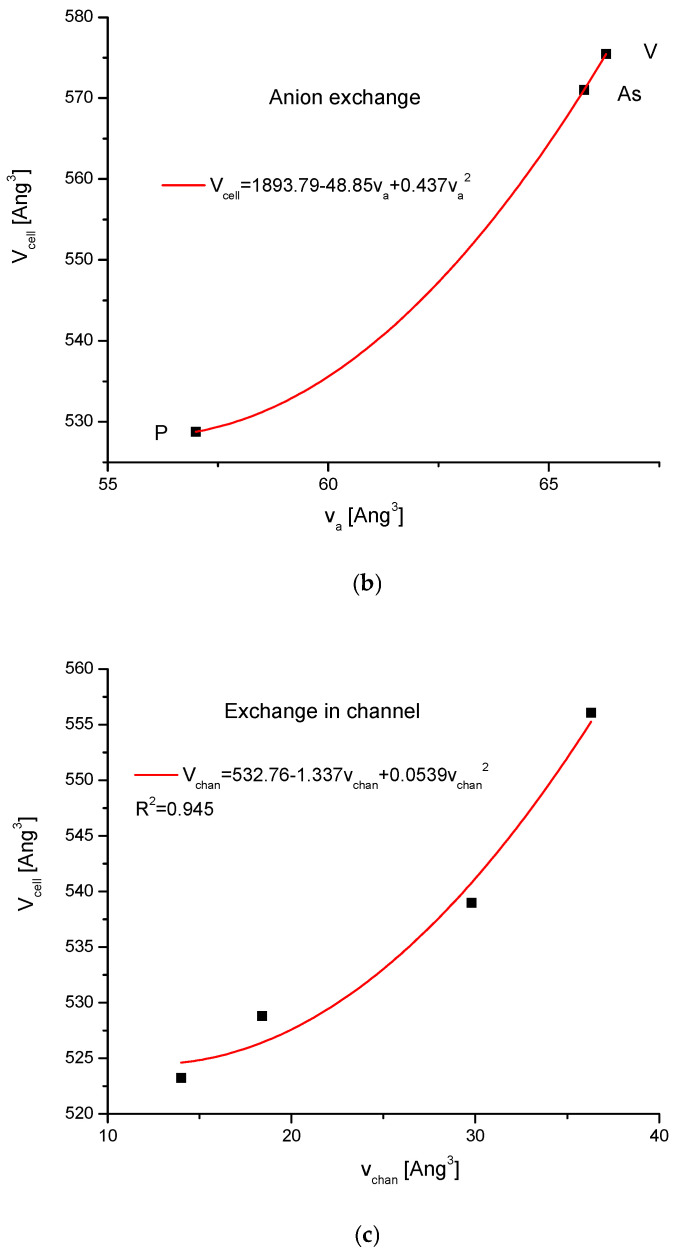

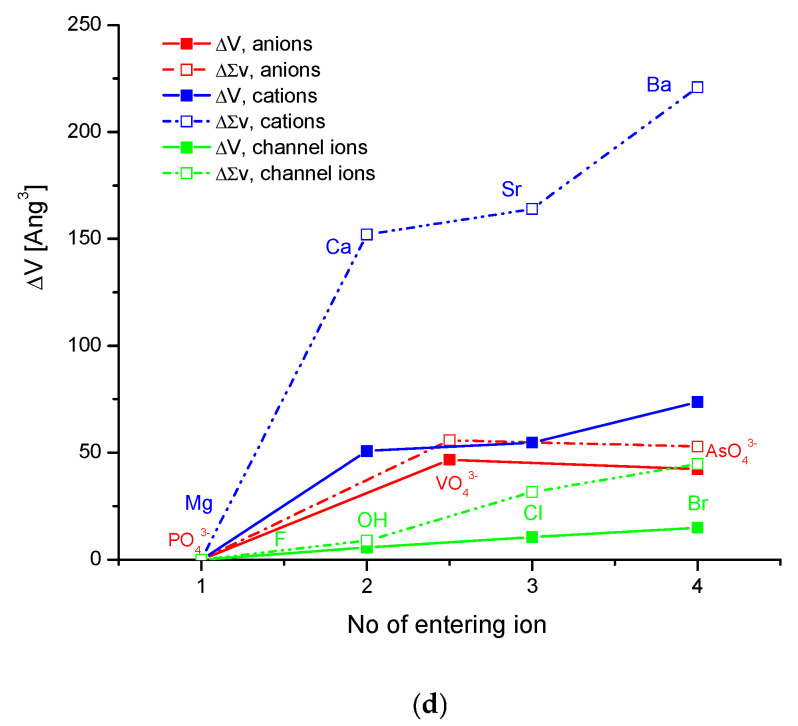
Crystallographic cell volume changes after the introduction of substituted ions in the positions of (**a**) main cation; (**b**) anion; (**c**) channel ion; (**d**) comparison of net real cell volume increase to the summary volume difference of substituted ions in relation to the original ones, ∑v.

It is interesting to trace how the total volume of the crystallographic cell changes together with the change of volumes of the introduced ions. The values of ion volumes are estimated mainly using thermochemical calculations. Still, the results are highly divergent among different authors and even in the different papers of the same team. In this situation, we decided to use the uniform results from one paper by Glasser and Jenkins [38] only, in order to avoid confusion. The base of the data is shown in Table 2. The results are shown in Figure 4a–c. As one could expect, the correlation is positive in each case—however, the results are not identical. All correlations are the polynomials of the second order.

In Figure 4a–c, we have presented the growths in the crystallographic cell volumes against the values of ion volumes. Each time we had positive second-order correlations with the increasing volumes of incoming ions. The next question is important: the cationic exchanges were most sensitive to the entering cation volume changes. The anionic changes occurred in a similar manner but in a more limited range. The most insensitive were channel exchanges since the relatively great changes of incoming species exerted only limited change in the cell volume. The volume of the channel was well-conserved in the cell.

The last effect can be observed quantitatively in Figure 4d. This time, the summary effect of the changes in the ionic volumes V − ∑v is presented on the abscissa, while the real number of the incoming ions is shown on the ordinate. One can see that the true growth in the cell volume was always lower than the total increase in volumes of the introduced ions in the cation, but it was less pronounced in the channel and especially in the anion locations. Nevertheless, there was a striking resemblance of the profile of the general cell growth to the summary profiles of the new located ions. It looked like the controlled relaxation of the crystal cell after the disturbance coupled with the ion exchange. To some extent, it resulted from the new interlocations of ions in the space and from the volume needed for two ion transfers in the space. This new additional space remained partially “frozen” after the act of the exchange. In general, the crystal swelled after the introduction of greater ions, but this effect was smaller than one would expect from the additional growth in the diameters/volumes of the introduced ions.

### 2.3. Vacancies

The notions of defects and vacancies arrived both in stoichiometric considerations concerning the course of the chemical reactions inside the apatites [39,40] and in the studies on their structures. Here, we are interested in the size of agglomerates of ions plus vacancies. Our interest relies on resolving the following questions:

- whether or not the apatites with the proven presence of vacancies belong to relevant isomorphic series;
- whether there is a possibility of finding the volume of the vacancy-ion agglomerate;
- whether the vacancy influences the behaviour of the rest of the apatite molecule.

Among the available data, we selected those which give the opportunity to extract some values concerning the volume of the vacancy. They concern a somewhat exotic series of lead apatites. The data are cited in Table 3.

**Figure 5:**
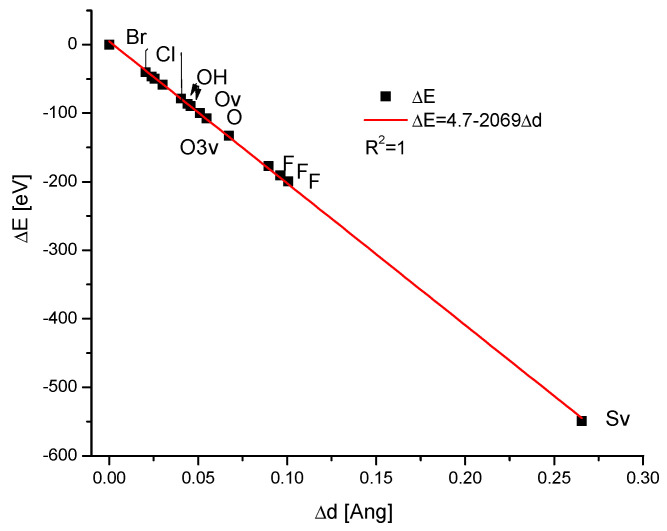
ΔE vs. Δd diagram for Pb-apatites. All of them belong to one isomorphic series with the same coefficients as, e.g., apatites from Figure 1a. Markers v denote the Pb-apatites with vacancies, as in Table 3.

At first, we established that all the samples from the above table belong to the isomorphic series of apatites. The confirmation is clearly shown by the straight line in diagram ΔE vs. Δd in Figure 5.

**Figure 6:**
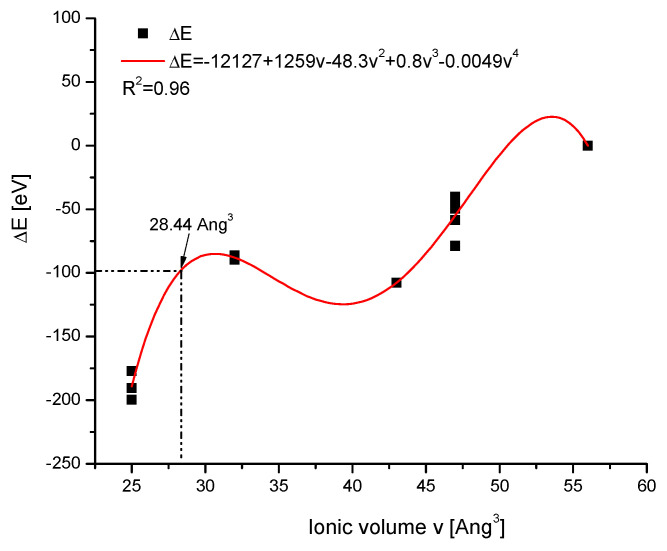
Relationship between the energy of ion exchange and the ionic volume of entering ions in the channel location. The arrow shows the predicted value of the volume for the agglomerate O-□.

Then, the diagram of the dependence of the energy of ion exchanges on the volume of entering ions was created from the data of Table 3, for which the known values of the ionic volumes were determined. This time, the relationship was somewhat more complicated, but it managed to approximate it by the polynomial of the fourth power with sufficient accuracy. In the next step, the relevant point was put on the curve (Figure 6), determining the postulated volume of the complex O□. It was 28.44 Å^3^. We can compare it with OH^−^—32, O^2−^—43, and O_2_^2−^—52 Å^3^ volumes. One can compare the results with the effects joining the introduction of real greater ions into the crystallographic set—it was always swelling. Here, for the defects, one observes the shrinking of the space covered by the agglomerate O□. Using the approximation given in Figure 6, one can determine the value 20.3 Å^3^ for the S□ agglomerate. It is interesting that Goldenberg et al. [51] estimated the volume of a single fragment of apatite channel as not greater than 30 Å^3^. One can set it with the values S^2^—67, SH—57 Å^3^. Each time, the significant shrinking of the agglomerates is noticeable in comparison with the real ion packing.

### 2.4. Temperature Changes of Hydroxyapatite Cells

**Figure 7:**
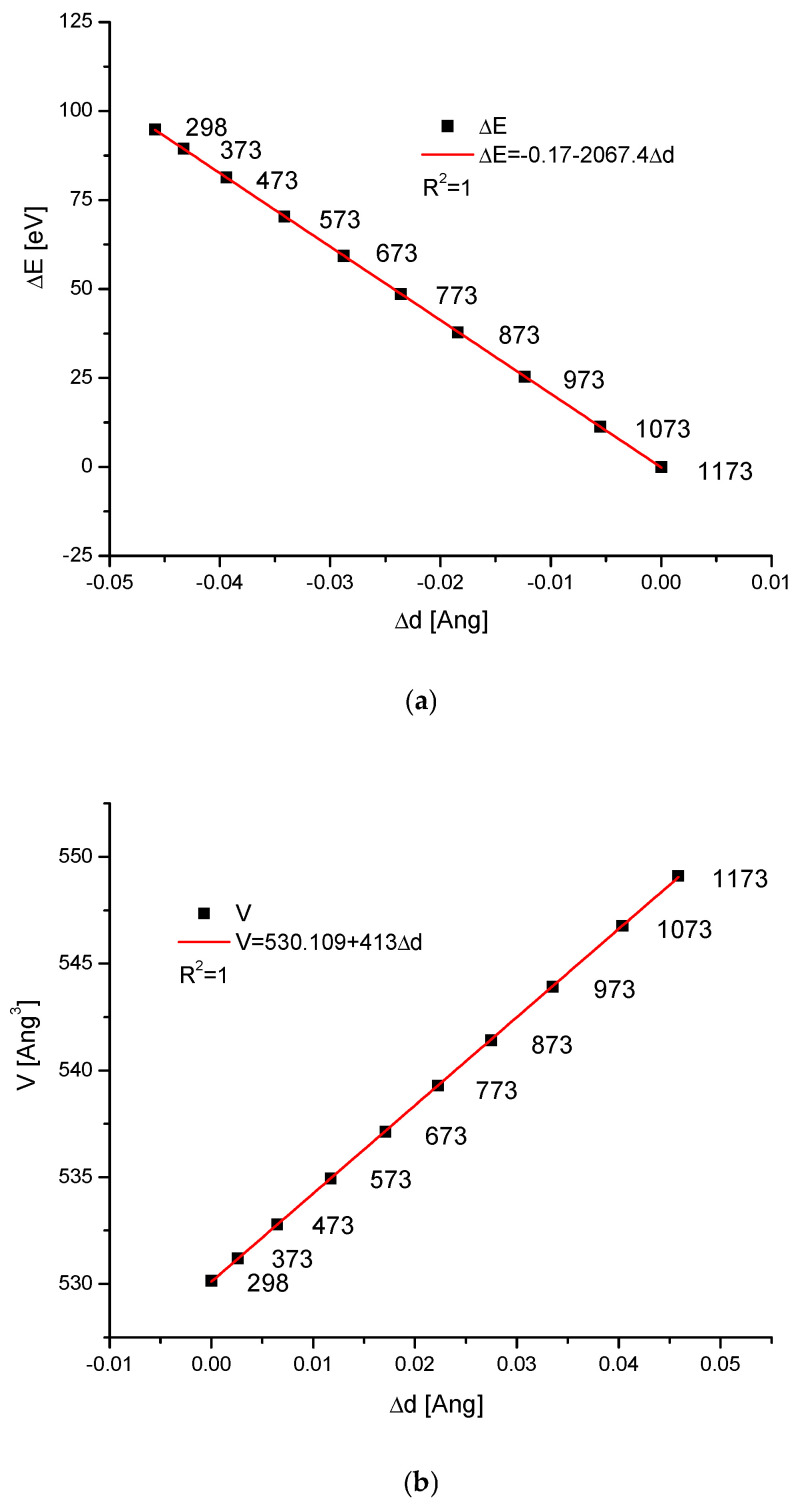

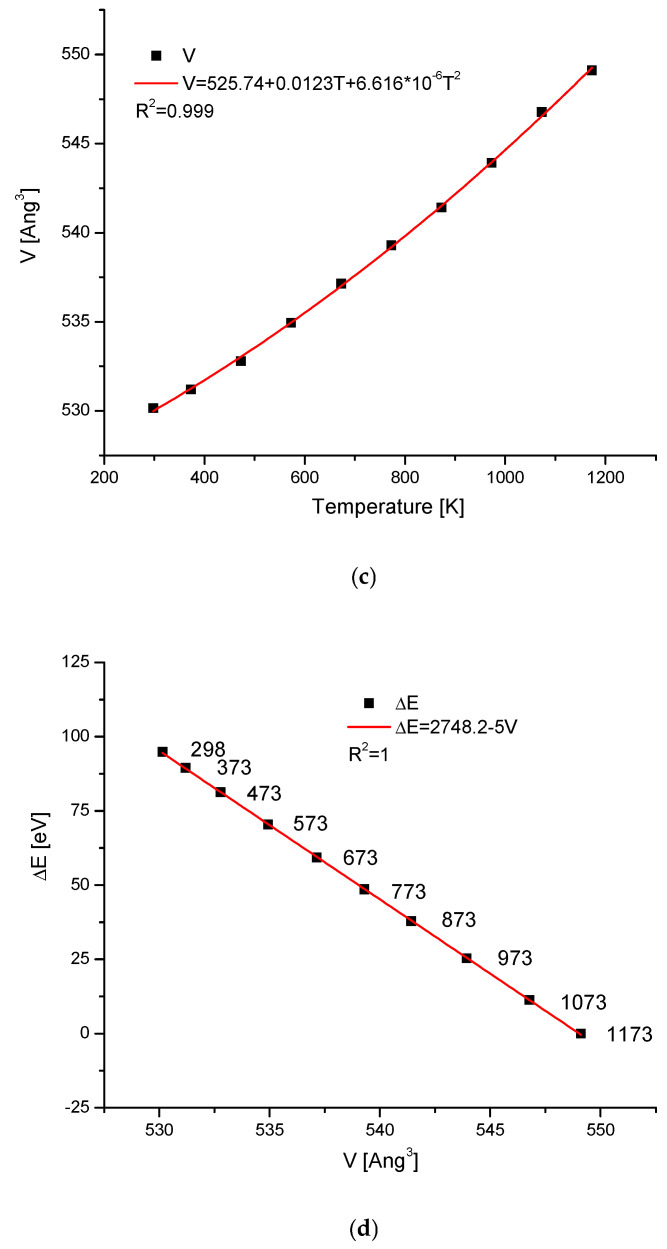

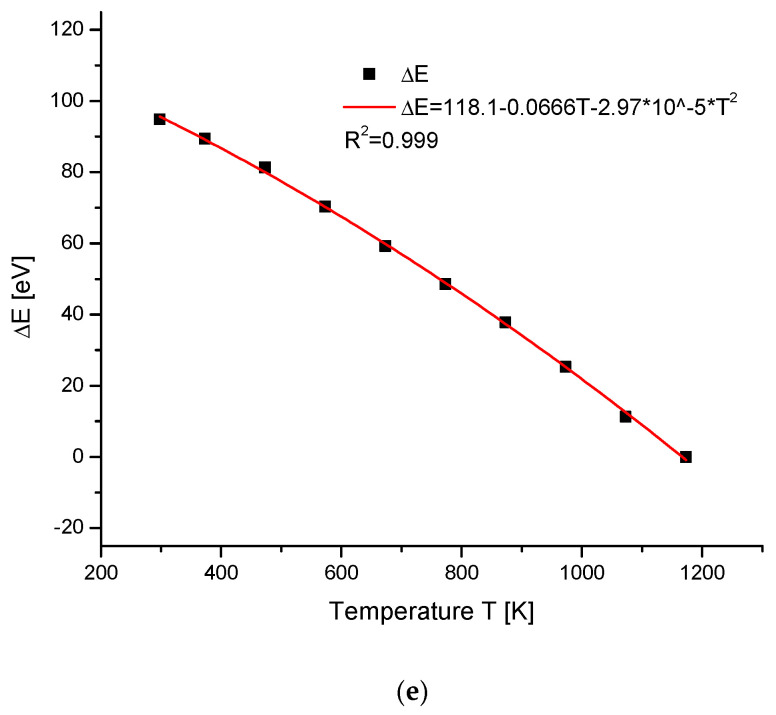
(**a**) Diagram ΔE vs. Δd for thermal action on hydroxyapatite; the numbers inside figures denote the relevant temperatures of measurements. (**b**) Coupling of the volume with the change of parameter d. (**c**) Growth of the volume by the influence of the temperature increase. (**d**) Straight line imagining the energy drop with the volume increase. (**e**) Coupling of energy change with the applied temperature.

We know that during temperature action on the hydroxyapatite, as well as on the other apatites, the crystallographic dimensions of the cells change. At the same time, the direction of changes is towards the sharpening of peaks in the spectra and more clear crystallisation. Sometimes, as a result of increasing temperature for different compounds, the phase change starts. It is interesting how the energy changes during these processes for the hydroxyapatite. We consider the case where the temperature action was observed and the resulting compounds were still located in the group of isomorphic chemicals. The data about the thermal expansion of hydroxyapatite were given in the paper by Knyazev et al. [52] (see Table 4). This situation is typical for the case without the phase change during the heating process. As one can observe, some results seem to be intuitive. For example, both the cell volume and the d parameter increased with the temperature growth (Figure 7b,c, respectively). One can even imagine that we could extrapolate the relevant values for the temperature of 0 K. In that situation, the ΔE value can be treated as the work of compression if one goes from higher to lower and lower temperatures (Figure 7a,d). It concerns the situations without phase transformations at a given range of temperatures. It is astonishing that both ΔE vs. Δd, ΔE vs. ΔV, and in this situation, ΔV vs. Δd relationships are all strictly linear ones.

## 3. Discussion

**Figure 8:**
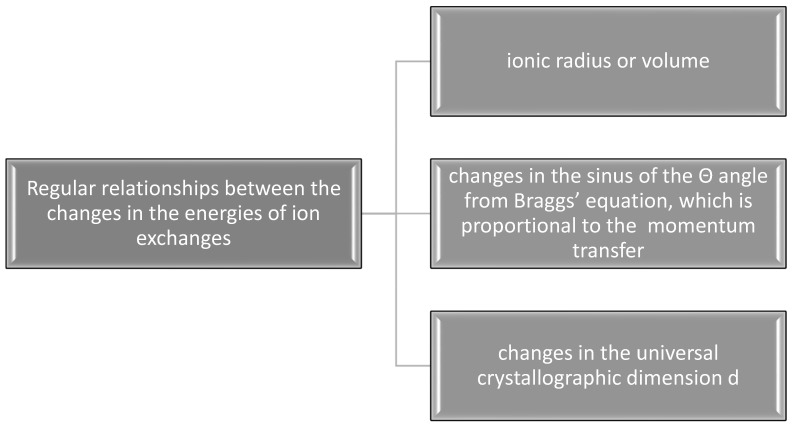
Expected regular relationships between the changes in the energies of ion exchanges (based on previous studies [19]).

It results from the consideration of hydroxyapatite structure that three different locations can be substituted in ion exchange processes—calcium cations, phosphate anions, and hydroxyl ions located in the channel position. This information implies that there should be three separate series of ion exchanges in apatite molecules, uncoupled with each other. As it was suggested in our previous position [19], one should expect regular relationships between the changes in the energies of ion exchanges and selected parameters (see Figure 8).

The arrival of the external ions in the hydroxyapatite structure occurred for the case of cations replacing Ca^2+^ in a very beautiful way—it is the strictly linear dependence for all the three mentioned cases against the volume of incoming ions vs. sinΘ and Δd (Figure 1). The involvement of anions, both the oxoanions in the positions of phosphate ion and simple anions in the channel location instead of hydroxyl ion, obeys the second-order polynomial equations against the ion volume values (Figure 2a and Figure 3a). Still, there were very regular dependencies. The first impression is that all the substitutions inside apatites occur in an extremely regular manner. The fact that the change of energy is coupled linearly with the ionic radii of entering cations is exciting (Figure 1a). One should rather expect the quadratic dependence, related to the cross-section of incoming ions. In Figure 2a and Figure 3a, we can observe the regular relationships, in this case, in relation to the volume of ions, and this dependence is the polynomial of the second order. The couplings look to be more dependent on the cross-section of the relevant ions than what was observed for the cations.

**Figure 9:**
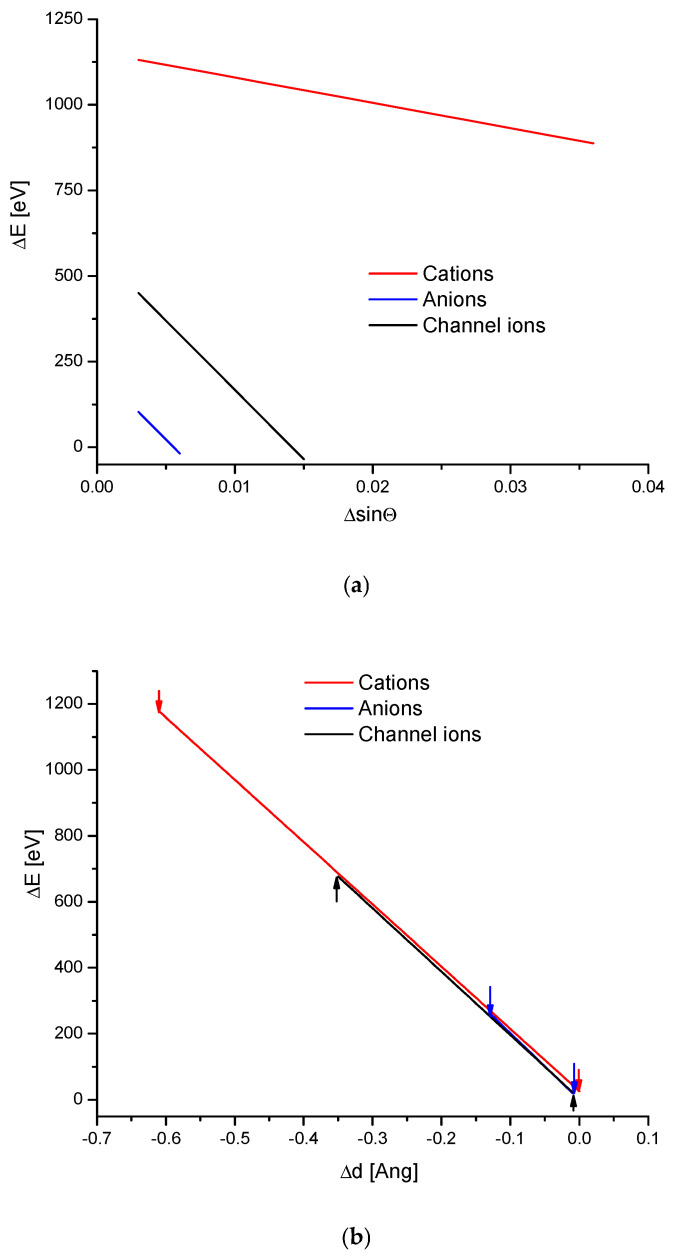
Consolidated sheet of the diagrams for three possible locations of ion exchanges: (**a**) ΔE vs. ΔsinΘ; (**b**) ΔE vs. Δd. Please note that the lines on the second diagram are nearly identical. The arrows show the boundaries of particular apatite ion exchange zones.

We have summarised the two types of dependencies of energy changes—on the ΔsinΘ value and on the Δd value (Figure 9). The former dependencies were rather divergent. More interesting is the relationship versus Δd. All the curves were superimposed. We earlier made the hypothesis that the equation

ΔE = kΔd(1f)

determines the belonging of the substance to its isomorphic series. In this aspect, all the enumerated ways of ion exchange in the apatites belong to the same type of reaction. There is no triple ion exchange in the apatites when it concerns the energy change against d change. It is shown in an illustrative way in Figure 9b.

Otherwise, such a conclusion may be drawn from the analysis of Equation (1). If one compares Equation (1c) with Equation (1f), then the following expression results:

k = −6.2/(d^2^sinΘ) (2a)

If the same is repeated for Equations (1e) and (1f), we have another expression for k:

K = −(1/6.2) × E^2^sinΘ (2b)

Please note that these expressions suggest the uniform k values for the three earlier expected forms of ion exchanges in apatites since they simply do not distinguish any selected ion exchange. The introduction of the relevant values gives in the first case the value of −2068.6 eV/Å, while in the latter case the value of −2069.2 eV/Å. If one wants to calculate the work necessary to increase the value of d(111) by the reasonable value of 0.1 Å, then for the case of cation exchanges, it will be −163.25 eV; for anions, −201.4 eV; for channel ions, −188.78 eV; for Pb apatites with the defects, −201.2 eV; for hydroxyapatites obeying the temperature treatment, −206.91 eV; and for the exchange of all cations, anions, and channel ions, −191.77 eV. It gives the average d-value −195.88 ± 15.06 eV. All this means the energy is liberated during the decompression of crystals.

**Figure 10:**
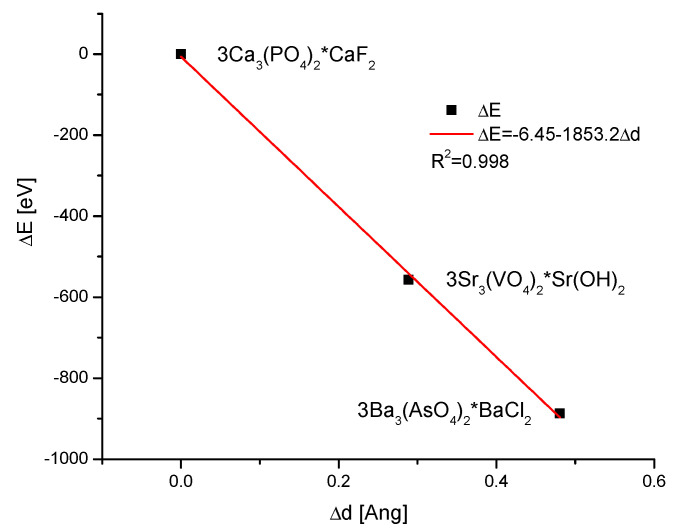
Diagram of ΔE vs Δd for the imaginably most divergent members of the family of apatites.

Another fact testifies to the universality of Equation (1f). If we imagine the wildest ionic exchanges inside the group of apatites, e.g., from 3Ca_3_(PO_4_)_2_∙CaF_2_ to 3Sr_3_(VO_4_)_2_∙Sr(OH)_2_ and finally to 3Ba_3_(AsO_4_)_2_∙BaCl_2_, where all the ions were exchanged step by step, we will still have the proper diagram ΔE vs. Δd (Figure 10).

The consideration of volumes—the first concerning the growing cell volumes and the other concerning the sum of volume differences of ions—introduced and expelled ones, testifies that it is a very clear excess of the summary volume differences of components over the gained volume of the cell (Figure 4d). It was valid, especially for cation exchanges and to a lesser degree for anion exchanges. Nevertheless, the patterns of the additional volumes were identical both for the volume gain of the crystal cell and the summary growth of the components.

Due to the handsome relationships between the energy change and the volume of incoming ions, there exists a possibility of determining the volume of agglomerates ion-□. The obtained values look reasonable.

A long time ago, it was observed that the heating of apatites improved the quality of X-ray diffraction spectra and the crystallinity of particles. We decided to study some effects of heating. The results show the linear dependence of energy change on the change in volume of crystallographic cells. It allows to join the energy change with the work of cell decompression during the heating. Moreover, one can establish the energy level of the crystallographic cells at the temperature of 0 K, if there is no phase transition at low temperatures. Here, in comparison with the situation at 1173 K, passing to the cell at the temperature 0 K demands storing 116,555 eV, being the energy of compression. The same concerns the cell volume at the temperature of 0 K, which is possible to be calculated according to the equation inside Figure 7c, and after the compression it is equal to a minimum value of 525.74 Å^3^.

### Future Directions

The important questions arriving from our approach are as follows:

- Is it possible to expand our method on other substances forming the series in other crystallographic systems, as e.g., the calcite series?
- Is it possible to split the somewhat strange results from Figure 9b on more detailed components?

The works concerning those questions are actually in progress.

## 4. Materials and Methods

### 4.1. Materials

The calculations are based on previously published reviewed manuscripts prepared by different authors in the available literature. For the content evaluation, we used keywords such as apatite, crystal structure, anion, cation, and substitution. Our remark: sometimes it is only a single available datum among the results in the world literature. It is presented in the tables in the text. The data were adapted to our aims, e.g., supplemented with additional information, e.g., about the crystallographic cell volume.

For the data search, we used platforms and databases such as Web of Science, Scopus, Scilit, and Google Scholar.

### 4.2. Methods

The important question is the calculation of the energies connected with the ion exchanges of different ions inside the crystallographic cell. The relevant equations were derived from Braggs’ equation, in the energy and not the wavelength representation. To derive them, one can imagine that some standard sample can be measured in position of Θ_1_ with the excitation energy E_1_, which means that the Braggs dimension is d_1_. Let us change the sample and then the measurement is in position Θ_2_, while the exciting energy is still E_1_ and new Braggs dimension is d_2_. Both situations are expressed by the relevant Braggs equations. One can ask what happens if we put the value Θ_2_ in the first equation. Then, we will have the new value of E_2_. Subtracting the left sides, we will have Equation (1a). It expresses the energy difference connected with the change of the crystal structure. The full set of possible-to-derive equations is as follows [19,53]:

ΔE = (6.2/d_1_)(1/sinΘ_1_ – 1/sinΘ_2_) (1a)

ΔE = (6.2/sinΘ_1_)(1/d_2_ − 1/d_1_) (1b)

ΔE = −6.2 × Δd/(d^2^ × sinΘ) (1c)

ΔE = −6.2 × Δ(sinΘ)/(d × sin^2^Θ) (1d)

ΔE = −(1/6.2) × Δd × E^2^ × sinΘ (1e)

The equations are equivalent to each other. The particular version of Equations (1c) and (1e) is the earlier applied empirical equation:

ΔE = kΔd(1f)

The meaning of the particular symbols are as follows:

E, ΔE—energy and the change of energy;
d, Δd—Braggs’ crystallographic dimension and its change;
Θ—the angle between the wave vector and the crystallographic plane.

For real crystals in a hexagonal system, with given a and c values, we can calculate d dimension from the equation:

1/d^2^ = 4/3[(h^2^ + hk + k^2^)/a^2^] + l^2^/c^2^ (3)

If we do it for the sample and the standard (the pure hydroxyapatite in this contribution), we can easily calculate Δd, and next ΔE, from Equation (1c) or Equation (1e).

## 5. Conclusions

Different ions enter the structure of hydroxyapatite in three different ways: either the cations can exchange Ca^2+^ ions or anions can exchange PO_4_^3−^ entities, or finally some simple anions can exchange OH^−^ ions in the channel position. As we have proved, such possibilities do exist. In our considerations, we restricted ourselves to the full exchanges only, limiting the list of all potential reactions. That those exchanges may be ordered in three systems was shown when one compared the dependence of exchanges on the ion radii/volumes, where it was relevant. Similarly, the relationships between the energy changes and corresponding changes in the values of the sinus of Θ angle showed the difference between momentum transfers for three kinds of exchanges. Nevertheless, the presentation of the energy changes as a function of the change in the value of the universal crystallographic dimension d reveals the uniformity of all ion exchanges inside the apatites. Thus, it is only one process of ion exchange in apatites, always demanding nearly the same energy change on the increment of the increase in generalised crystallographic dimension d. This dimension d is of the universal meaning for the isomorphism of apatites. Our way of reasoning helps to determine the radius of the agglomerate ion-□ (vacancies). Finally, we have determined the thermal relationship between the ΔE − Δd − T(K). It leads to the conclusion that the unit cell volume and parameter d have the minimum values at the temperature of 0 K, while the stored energy of the crystallographic compression is at maximum.

## Figures and Tables

**Table 1:** Basic data concerning the studied apatites for the ion exchanges of cations, anions, and anions in the channel location. ICDD means The International Centre for Diffraction Data, with the number of the relevant standard.

Kind of Ion Exchange	Formula	a (Å)	c (Å)	a/c	Ionic Radii (pm)/Volumes (Å^3^)	References
Cationic	Ca_10_(PO_4_)_6_(OH)_2_	9.418	6.884	1.368	114	ICDD 09-0432
	Mg_10_(PO_4_)_6_(OH)_2_	8.722	6.624	1.317	86	[20]
	Sr_10_(PO_4_)_6_(OH)_2_	9.777	7.288	1.342	132	[21]
	Ba_10_(PO_4_)_6_(OH)_2_	10.1901	7.7212	1.320	149	[22]
	Pb_10_(PO_4_)_6_(OH)_2_	9.8663	7.4262	1.329	133	[23,24]
	Cd_10_(PO_4_)_6_(OH)_2_	9.3352	6.6643	1.401	109	[25]
Anionic	Ca_10_(PO_4_)_6_(OH)_2_	9.418	6.884	1.368	90	ICDD 09-0432
	Ca_10_(VO_4_)_6_(OH)_2_	9.7405	7.0041	1.391	83	[26]
	Ca_10_(AsO_4_)_6_(OH)_2_	9.7156	6.9857	1.391	88	[27]
	Ca_10_(CrO_4_)_6_(OH)_2_	9.683	7.010	1.381	97	[28]
Anionic in the channel	Ca_10_(PO_4_)_6_(OH)_2_	9.418	6.884	1.368	32	ICDD 09-0432
	Ca_10_(PO_4_)_6_F_2_	9.3684	6.8841	1.361	25	ICDD 15-0876
	Ca_10_(PO_4_)_6_Cl_2_	9.5903	6.7666	1.417	47	[29]
	Ca_10_(PO_4_)_6_Br_2_	9.7611	6.7391	1.448	56	[30]
	Ca_10_(PO_4_)_6_O	9.432	6.881	1.371	43	[31]
	Ca_10_(PO_4_)_6_S	9.455	8.84	1.070	67	[32]
	Ca_10_(PO_4_)_6_Se	9.5007	6.8406	1.339	181	[33]

**Table 2:** Data concerning the volumes of apatites and volumes of incoming ions.

Kind of Ion Exchange	Formula	a (Å)	c (Å)	Cell Volume V (Å^3^)	Ionic Volumes V (Å^3^)	References
Cationic	Ca_10_(PO_4_)_6_(OH)_2_	9.418	6.884	528.8	20.1	ICDD 09-0432
	Mg_10_(PO_4_)_6_(OH)_2_	8.722	6.624	436.4	4.9	[34]
	Sr_10_(PO_4_)_6_(OH)_2_	9.777	7.288	603.3	21.3	[30]
	Ba_10_(PO_4_)_6_(OH)_2_	10.1901	7.7212	694.3	27	[31]
Anionic	Ca_10_(PO_4_)_6_(OH)_2_	9.418	6.884	528.8	57	ICDD 09-0432
	Ca_10_(VO_4_)_6_(OH)_2_	9.7405	7.0041	575.5	66.3	[29]
	Ca_10_(AsO_4_)_6_(OH)_2_	9.7156	6.9857	523.2	65.8	[28]
Anionic in the channel	Ca_10_(PO_4_)_6_(OH)_2_	9.418	6.884	528.8	18.4	ICDD 09-0432
	Ca_10_(PO_4_)_6_F_2_	9.3684	6.8841	523.2	14	ICDD 15-0876
	Ca_10_(PO_4_)_6_Cl_2_	9.5903	6.7666	539.0	29.8	[30]
	Ca_10_(PO_4_)_6_Br_2_	9.761	6.739	556.1	36.3	[30]

**Table 3:** The basic crystallographic data about lead apatites.

Kind of Ion Exchange	Formula	a (Å)	c (Å)	Ionic Radii (pm) /Volumes (Å^3^)	References
Channel exchanges	Pb_10_(PO_4_)_6_O	9.826	7.431	43	[41]
	Pb_10_(PO_4_)_6_O□	9.84	7.43		[42]
	Pb_10_(PO_4_)_6_S□	9.45	6.84		[43]
	Pb_9_□(PO_4_)_6_O□_2_	9.826	7.357		[44]
	Pb_10_(PO_4_)_6_OH_2_	9.866	7.426	32	[45]
	Pb_3_(PO_4_)_2_	9.826	7.357		[44]
	Pb_10_(PO_4_)_6(_(OH)_2_	9.8612	7.4242	32	[30]
	Pb_10_(PO_4_)_6_Br_2_	10.0618	7.3592	56	[30]
	Pb_10_(PO_4_)_6_Cl_2_	9.9767	7.3255	47	[30]
	Pb_10_(PO_4_)_6_Cl_2_	9.9764	7.3511	47	[46]
	Pb_10_(PO_4_)_6_Cl_2_	9.95	7.31	47	[47]
	Pb_10_(PO_4_)_6_Cl_2_	9.993	7.334	47	[48]
	Pb_10_(PO_4_)_6_Cl_2_	9.9981	7.344	47	[49]
	Pb_10_(PO_4_)_6_F_2_	9.7547	7.2832	25	[30]
	Pb_10_(PO_4_)_6_F_2_	9.777	7.310	25	[50]
	Pb_10_(PO_4_)_6_F_2_	9.760	7.300	25	[51]

□—vacancies.

**Table 4:** Data about the thermal expansion of hydroxyapatite.

Compound	Temperature (K)	a (Å)	c (Å)	V (Å^3^)	References
Ca_10_(PO_4_)_6_(OH)_2_	298	9.4273	6.8882	530.2	[53]
	373	9.4354	6.89	531.2	
	473	9.444	6.898	532.7	
	573	9.4589	6.904	535	
	673	9.4727	6.9122	537.15	
	773	9.4833	6.9245	539.3	
	873	9.4986	6.9294	541.43	
	973	9.514	6.939	544	
	1073	9.53	6.952	546.8	
	1173	9.54	6.967	549.1	

## Data Availability

The original contributions presented in this study are included in the article. Further inquiries can be directed to the corresponding author.
